# Temporal trends in incidence, recurrence and prevalence of stroke in an era of ageing populations, a longitudinal study of the total Swedish population

**DOI:** 10.1186/s12877-019-1050-1

**Published:** 2019-02-04

**Authors:** Karin Modig, Mats Talbäck, Louise Ziegler, Anders Ahlbom

**Affiliations:** 1 Institute of Environmental Medicine, Unit of Epidemiology, Karolinska Institutet, Box 210, SE-171 77 Stockholm, Sweden; 2 Institute of Environmental Medicine, Unit of Epidemiology, Karolinska Institutet, Stockholm, Sweden; 3 Department of Clinical Sciences, Danderyd Hospital, Karolinska Institutet, Stockholm, Sweden; 4 Institute of Environmental Medicine, Unit of Epidemiology, Karolinska Institutet, Stockholm, Sweden; 5 Centre for Occupational and Environmental Medicine, Stockholm County Council, Stockholm, Sweden

## Abstract

**Background:**

Stroke incidence has declined during the past decades. Yet, there is a concern that an ageing population together with improved survival after stroke will result in a raised proportion of the population who have experienced a stroke, as well as increasing incidence rate of recurrent strokes, and, absolute numbers of strokes. The objectives of this study were to investigate how the age specific incidence rates of recurrent strokes have developed in relation to the incidence rates of first strokes and how the postponement in age look like, and to see how the prevalence proportion of stroke as well as the absolute number of incident strokes has changed over time.

**Methods:**

This study includes the total Swedish population born 1890–1954 living in Sweden from 1987. Stroke was identified through hospital admissions and deaths in national health registers (mandatory for all hospitals in Sweden). Age specific incidence rates were calculated for first, second, all recurrent, and all strokes for each calendar year between 1994 and 2014 for each age between 60 and 104 years. The proportion in the population with a history of stroke up to 7 years back in time was also calculated for different age groups and for different calendar years.

**Results:**

Not only the incidence rate of first stroke but also of recurrent strokes have declined. The declines are evident in all ages up to 90 years of age, but not in ages above 90 years. Despite improved survival in stroke, the prevalence proportion has declined over the period and was around 3% in 2014 (somewhat higher for men than women). Even incident cases of stroke in absolute number has declined.

**Conclusions:**

Decreasing incidence rates of stroke have offset an increase in both absolute and relative numbers of stroke that otherwise would have taken place due to improved survival and an ageing population. The decline in stroke recurrence has been as strong as the decline in first strokes.

**Electronic supplementary material:**

The online version of this article (10.1186/s12877-019-1050-1) contains supplementary material, which is available to authorized users.

## Background

Because populations are becoming older and survival from many diseases, including stroke, has improved over the past decades there is concern that the disease burden in general will increase. Stroke is one of the diseases that has caused such concern [1, 2]. One reason is that the absolute number of stroke events may increase due to demographic changes even if age specific incidence rates come down. Another reason is that the rate of recurrent stroke may increase when a larger proportion survive their first stroke, since survivors have an increased risk of subsequent strokes as compared to the general population [2, 3]. About 25% of all strokes are recurrent and not first strokes.

Even if incidence rates for stroke have come down, at least in the population 65 years and above, where the vast majority of stroke cases occur [4, 5], a previous study projected that because of ageing, the absolute number of incident stroke cases will increase dramatically in Sweden in the coming years [6]. A review of European studies further discusses the problem of increasing prevalence of stroke as the elderly population grows larger, incidence stays stable and survival improves [2]. Also the World Health Organization projects the burden of stroke to increase to year 2025 [7] stating that “even if the incidence rate of stroke is declining among the old, the absolute number of stroke cases is expected to increase dramatically in coming years because of the ageing of the population, since the incidence rate of stroke is closely related to age”. The report further discuss that stroke survivors are at a high risk for recurrent strokes as well as for other adverse health outcomes.

Considering the concern that is raised about an increased burden from stroke when populations age, we find the literature sparse with regards to studies examining recent trends of incidence rates of stroke, including recurrent strokes, and prevalence proportions of stroke, especially including older ages. Stroke recurrence is commonly calculated as a proportion of the stroke survivors experiencing a second stroke within a certain time period (most often one year) [3, 8–11]. Although this is interesting, it is not precise enough to capture shifts in time. For example that individuals experience a recurrent stroke after the defined period of follow up, or if they experience more than one recurrent stroke (the latter of importance to capture any potential increase in absolute number of strokes). Further, among studies estimating stroke recurrence only a few present time trends. However, of the relatively few studies we found, all presented a decline over time in the proportion of patients experiencing a second stroke [8, 9, 12]. Only one of the studies investigated the change in risk of a recurrent stroke beyond a year from the first stroke.

In order to better understand the future burden of stroke on society this study aims at: first, to investigate how the age specific incidence rates of recurrent strokes have developed in relation to the incidence rates of first strokes and how the postponement in age look like. Second, to see how the prevalence proportion of stroke as well as the absolute number of incident strokes has changed over time.

Disentangling this will help to make a better and more clear statement about how the burden of stroke has developed during the past two decades in Sweden, and some indications of what is to be expected for the future.

## Methods

### Material

We drew on national population registers [13–15] identifying all individuals born 1890–1954 living in Sweden during 1st of January 1987 and 31st of December 1993. Subjects were followed from 1st of January 1987 for stroke, death and emigration until end of follow-up on 31st of December 2014. The population was identified from the Total Population Register held by Statistics Sweden [13]. Stroke events were identified by combining hospital admissions with a stroke diagnosis through the National Patient Register (NPR) [14] and death from stroke from the Cause of Death Register (CDR) [15]. The International Statistical Classification of Diseases and Related Health Problems (ICD) codes were used for identification of stroke, using codes 431, 434, 436 (ICD-9) and I61, I63 and I64 (ICD-10). Stroke was identified by using the main diagnoses of inpatient care in the NPR or from the underlying cause of death in the CDR. A restriction was also set so that individuals had to be hospitalized at least one night to avoid misclassification of suspected stroke not turning out to be stroke and therefore discharged the same day from hospital. However, the patient register has shown to have high specificity and sensitivity with regards to stroke [14]. All analyses were additionally performed separately for ischemic (ICD-9: 434 and ICD-10: I63) and haemorrhagic (ICD-0: 431 and ICD-10 I61) stroke.

### Analyses

In order to estimate the incidence of first stroke, information about the entire disease history of individuals would be required. When using administrative health registers there is a time point when the register starts (here 1987) resulting in left truncation, i.e., before a certain time there is no information about the disease history of individuals, meaning that it is not possible to definitely define a first occurrence of disease. A practical way to handle this is to apply a wash out period at start where events are disregarded and only individuals free of disease after the wash out period are followed up. As far as we know, there is no golden standards for how to define a first stroke using administrative registers. We applied a 7-year period corresponding to the Swedish National Board of Health and Welfare for their statistics of incidence of first myocardial infarction and stroke [16, 17]. A first stroke was thus defined as any hospital admission for stroke or death from stroke occurring after a 7-year period free of hospital admissions for stroke. The population at risk was calculated as the total person time in days until first stroke or censoring.

A recurrent stroke was defined as a hospital admission for stroke, or death from stroke, occurring at least 28 days after a previous hospital admission for stroke. The population at risk for recurrent stroke consisted of everyone who had had a previous stroke within the past 7 years, but not currently (within the 28 days) experiencing a stroke. We estimated both incidence rate of the first recurrent stroke, that is; the second stroke, as well as all recurrent strokes regardless of order.

### Calculation of incidence rates

Age specific incidence rates were calculated for men and women separately for first, second, all recurrent, and all strokes combined for each year between 1994 and 2014 for individuals between 60 and 104 years old. The number of cases for each age (attained age) and calendar year was divided by the corresponding number of person years at risk. For incidence rate of first stroke the denominator consisted of the total person time of those who had not experienced a stroke within the past 7 years. For incidence rate of the second stroke the denominator consisted of the total person time of those who had experienced one previous stroke the past 7 years. For all recurrent strokes the denominator consisted of the total person time of those having experienced a previous stroke, regardless of the number of previous strokes. Finally, in addition to the first, second and all recurrent strokes, we estimated the incidence rate of all strokes combined, over the total person time in the population.

### Calculation of prevalence proportion

The proportion in the population alive with a history of stroke up to 7 years back in time, was calculated on December 31st each calendar year. This prevalence proportion is presented for a selection of calendar years to see how the proportion changed over time, as well as for different age groups in 2014 to see how the prevalence proportion differed by age.

### Absolute numbers

In addition to the incidence rates and the prevalence proportion, we calculated the absolute number of incident strokes over the study period, that is, all strokes occurring at least 28 days apart.

## Results

The results are based on a total of 19,467,912 person years for men and 23,860,484 person years for women. In total 282,769 strokes occurred among men and 316,067 among women over the study period 1994 to 2014.

**Fig. 1:**
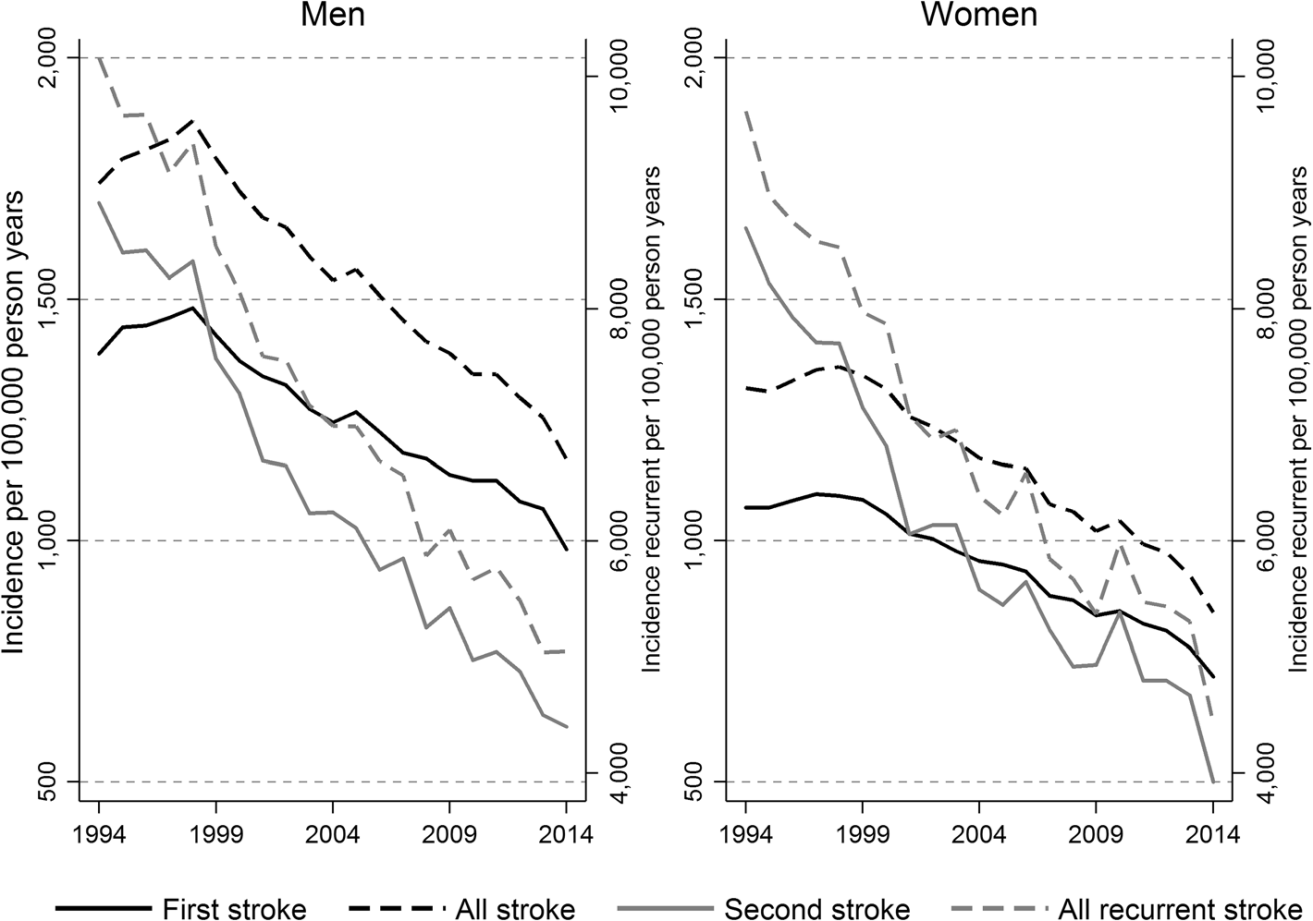
Incidence rate of first and all strokes (values on left Y-axis) and incidence rate of second and all recurrent strokes (values on right Y-axis). Values presented over the period 1994 to 2014 for men and women aged 60–104 (age adjusted)

Figure 1 displays the trend in incidence rates for first strokes, all strokes, second strokes and all recurrent strokes. All incidence rates have decreased over the study period and the pattern is similar for men and women. During the first 4–5 years, however, there was a slight increase in the incidence of first and all stroke, primarily for men. Incidence rate of first and second stroke is higher for men compared to women, even if the gender difference is larger for first stroke than for the second, 982/100000 (men) vs 719/100000 (women) for first stroke in 2014, and 4401/100000 (men) vs 3923/100000 (women) in 2014. The graph (Fig. 1) presents an average for the ages 60–104 (age adjusted). Looking more closely into age groups it turns out that the declines in incidence rates can be observed in ages up to 90 years but not in ages above that (see Additional file 1: Figure S1a-h showing age specific results).

**Fig. 2:**
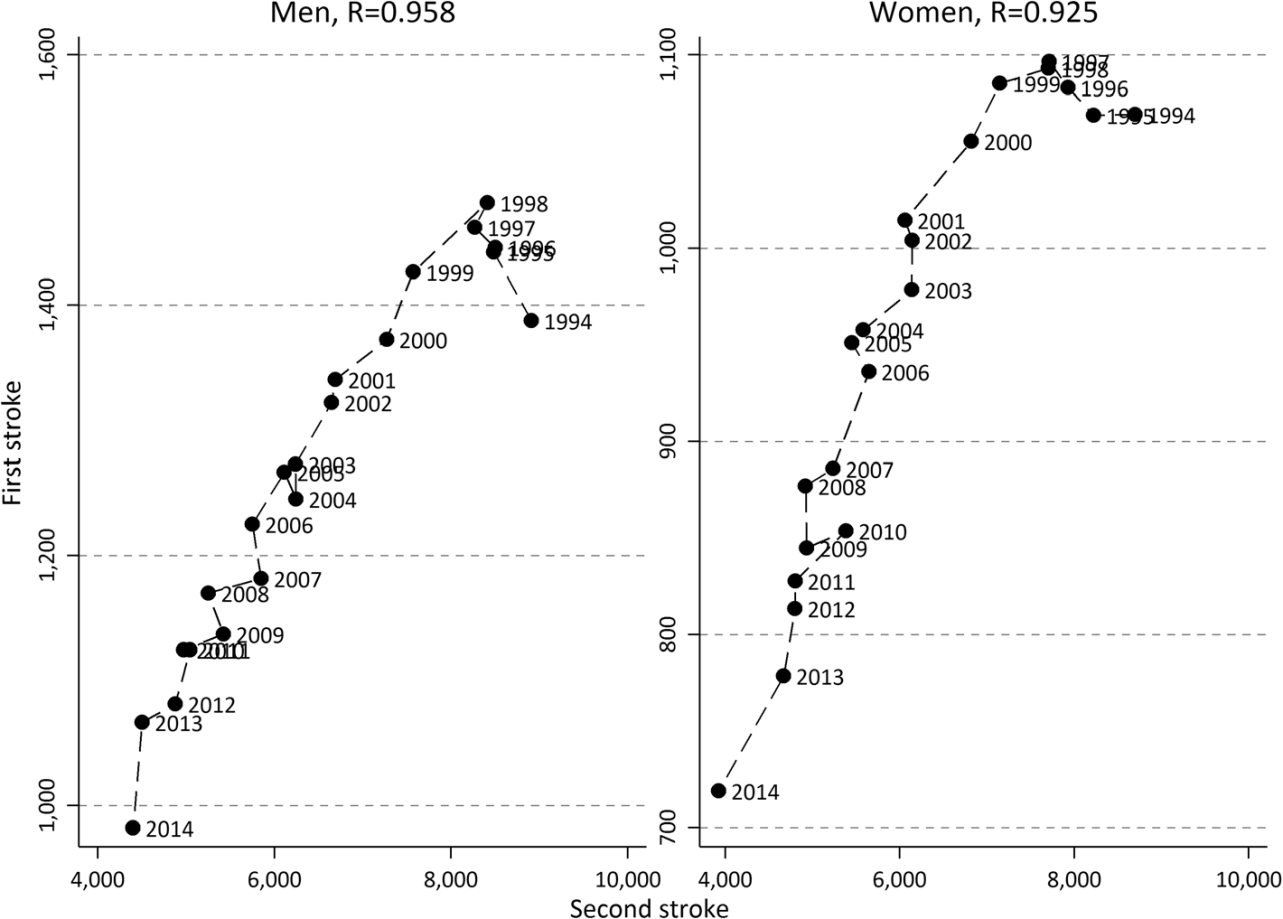
Incidence rate of first stroke (y-axis) and second stroke (x-axis) for men and women aged 60–104 (age adjusted), and correlation coefficients, between 1994 and 2014

Another way to analyse if the incidence rate of a second stroke has declined in parallel with the incidence of first strokes is to plot the decline in the first stroke on the Y-axis and the second stroke on the X-axis, which is presented in Fig. 2. The linear decline suggests that the decline in second strokes follows the decline in first strokes, with the exception of the first five years where the incidence of first stroke is increased. The correlation of the declines in first and second strokes is very high, 0.96 for men and 0.93 for women. Looking into age groups the linear fashion can be observed up to ages around 85. After this age there is no clear pattern that the declines in incidences follow each other (results not presented but available upon request from the authors, also supported from Additional file 1: Figure S1a-h).

**Fig. 3:**
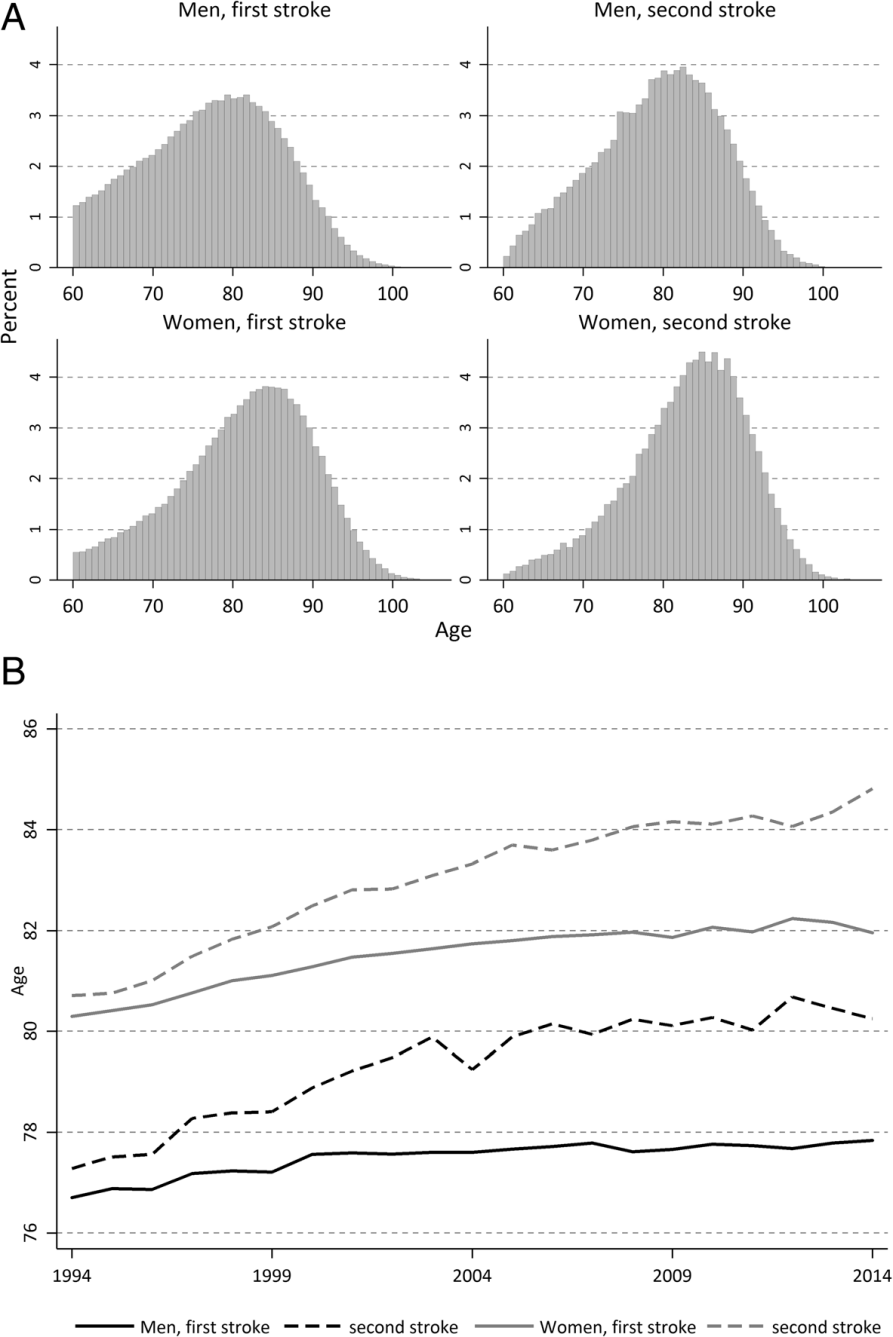
a Age distribution of first and second stroke 1994–2014. Men and women. b Mean age of first and second stroke over the period 1994 to 2014. Men and women

A decline in incidence rate stems from either a postponement of the disease to higher ages, or from the risk of disease at each age is getting lower. In Fig. 3 a) and b) we can see the age distribution of first and second stroke for men and women, as well as the change in mean age over the period. For first stroke, there has been a postponement of 1 year for men and 1.5 years for women. For the second stroke, the postponement is larger, 3 years for men and 4 years women. Even if some of the postponement for the second stroke is coming from the postponement of the first, there is a larger postponement in age for the second stroke than for the first.

**Fig. 4:**
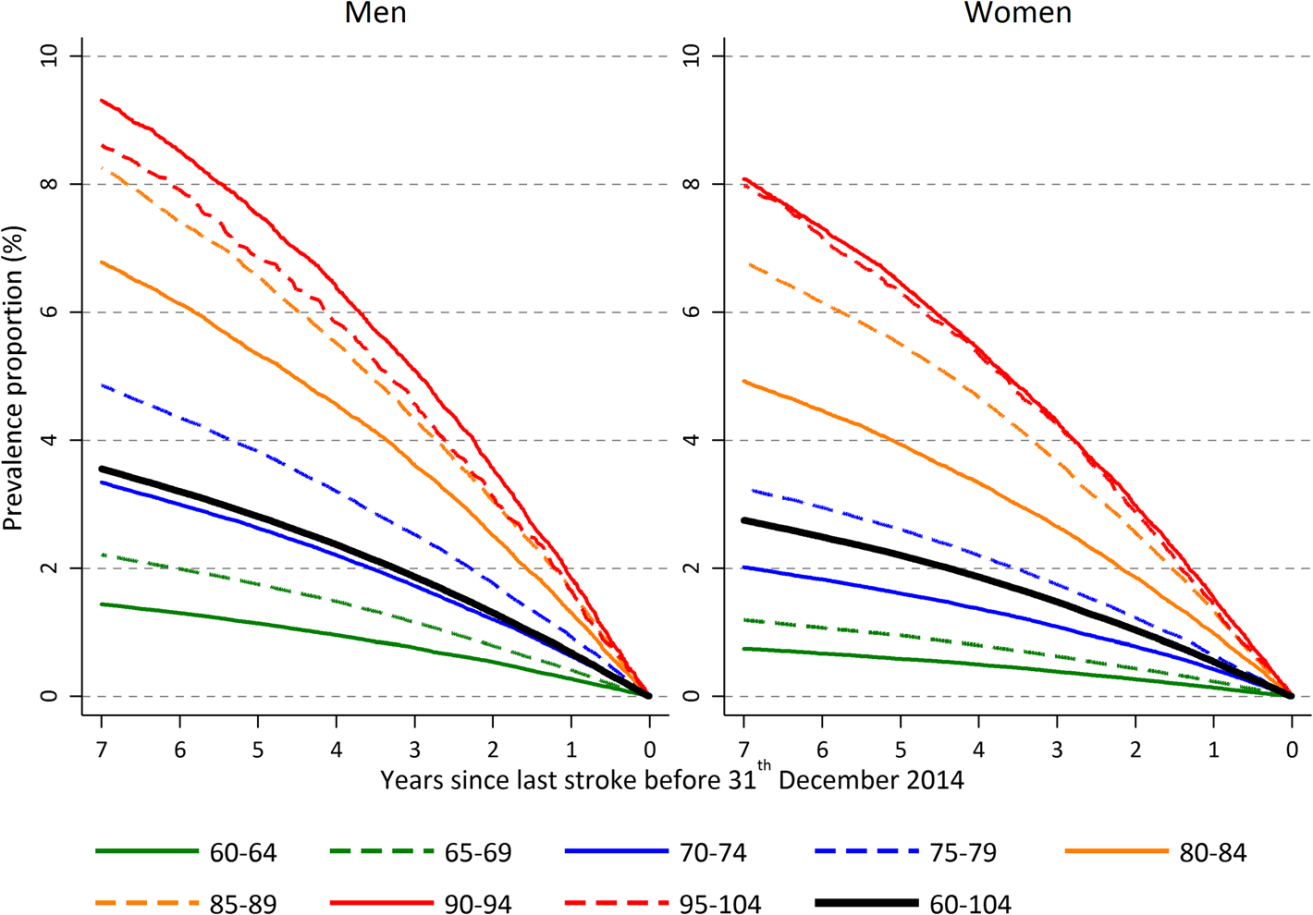
Proportion of the population alive 31st of December 2014 with a history of stroke up to 7 years back in time, for different age groups. Men and women

**Fig. 5:**
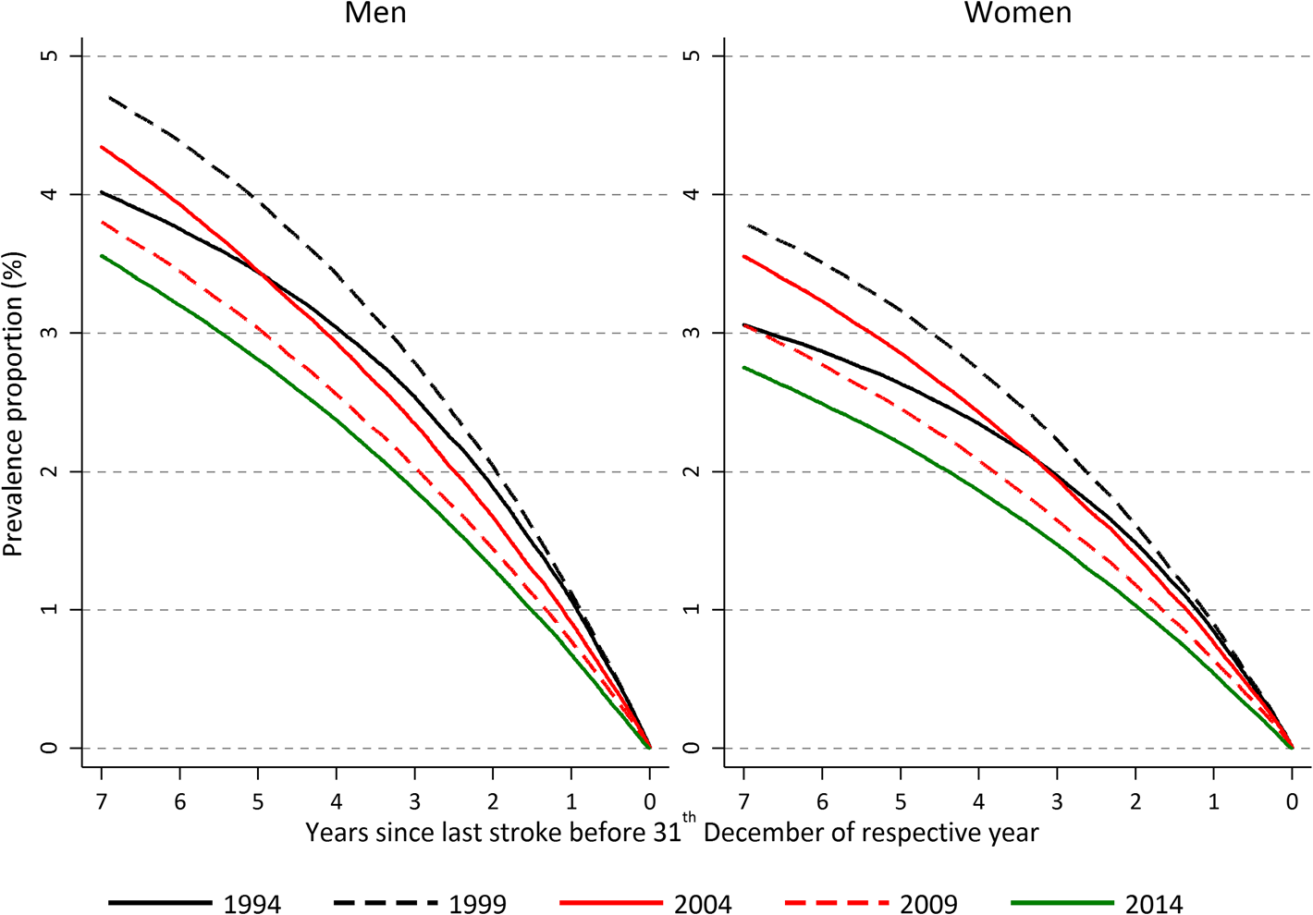
Proportion of the population alive 31st of December with a history of stroke up to 7 years back in time, for 5 different calendar years, ages 60 to 104 years. Men and women

Figure 4 displays the proportion in the population alive at December 31st 2014 with a history of stroke up to 7 years back in time for different age groups. This prevalence proportion increases with age and range from 1.5% for males and 1% for females in the 60–64 age group to 9% for males and 8% for females in the 95–104 age group. Restricting to the proportion alive with a history of stroke 1 year back in time, gives a proportion of less than 0.5% for the youngest age group and around 2% for the oldest. Figure 5 displays how the prevalence proportion has changed over time in the population. As in the previous figure, the prevalence proportion is calculated up to 7 years back in time, but now for 5 different calendar years. In the first period, from 1994 to 1999 the prevalence proportion increased, and since then it has decreased. That is, in 2014 there were slightly less than 3% of the women in the ages 60–104, and among men a little higher 3.5%, who had experienced a stroke any time during a 7-year period back in time. In 1999 these figures were 4.7% among men and 3.8% among women. Also the absolute number of strokes declined over the study period (see Additional file 2: Figure S2).

In complimentary analyses, we analysed ischemic and haemorrhagic stroke separately. The trends for both incidence rates (Additional file 3: Figure S3) and prevalence proportions (not shown but available from the authors upon request) look very similar for both stroke subtypes.

## Discussion

There is concern that the ageing of the population and the improved survival with stroke might result in increasing incidence rates of recurrent strokes and in the proportion of people with a history of stroke [1, 2, 6, 7]. However, our results show that, at least during the last decades in Sweden, the decreasing incidence rates of stroke (first as well as recurrent) have been large enough to counter this development. The prevalence proportion of stroke has not increased. Neither has the absolute number of incident stroke cases. The lack of improvements in ages 90+ is, however, an important observation. Since this age group will increase in size in the future, it may affect the prevalence proportion upward, since around 16% of all first strokes and 20% of all recurrent strokes occur in this age group (in 2014).

The postponement in age was larger for the second stroke than for first stroke. This suggests that for first strokes, the decline in incidence rate is to a higher extent explained by fewer individuals attaining a stroke at each age than for recurrent strokes. For recurrent strokes, the decline in incidence is explained by a postponement of the stroke to a higher age. This could be interpreted as if primary prevention manages to prevent some of the first strokes while the secondary prevention to a higher extent manages to postpone the second stroke. Further, this observation calls for caution when estimating risk of recurrent stroke as the proportion of patients experiencing a second stroke within a given time period; the time period may have been extended but not the proportion as such.

The results from the current study are in line with previous studies which also found declining incidence rates for first strokes [4, 5, 9, 18], and a few also for recurrent strokes [8, 9, 19], even if the latter did not estimate incidence rate of recurrence but the proportion of individuals who had experienced a first stroke that got another stroke within one year [8]. Such proportion is a clinically relevant perspective on stroke recurrence, but from a population perspective, long-term trends of incidence rates provide a more thorough understanding of how disease burden of stroke looks like in the population as a whole. Our result contributes with information that the incidence rates of both first and recurrent strokes have declined in parallel, and we present age specific results also for those above 85 years.

As for the level of incidence rate of stroke, it varies between studies due to various definitions of stroke (first, all etc.). A review study from 2011 compare incidence rates for stroke from several European countries and find large differences. The incidence rate is for example more than twice as high in Germany than in Spain or the United Kingdom [20]. Our rates are even higher, but this is likely because we study the population aged 60 years and older whereas most of the studies in the review include all ages, which will create a lower incidence rate because of a larger population at risk in relation to cases. In studies of incidence rate, it is essential that if only first strokes are in the numerator, the rates cannot be directly compared with rates of all strokes [21].

Our results of declining incidence trends are further in line with those presented in a report from the nationwide Swedish Stroke Register, Riksstroke [22, 23]. One of the main reasons for the declining incidence rates of first stroke could be the halved smoking prevalence until 2014, from just above 20% in the mid-nineties. From an international perspective, this is a large decrease and could be part of an explanation to the larger decrease in incidence in Sweden as compared to some other developed countries. Moreover, in the same period, a more aggressive primary preventive treatment of hypertension became part of the standard clinical practice in Sweden as in the rest of the Western world. Cerebrovascular disease is known to be strongly associated with hypertension [24] why this prevention strategy is likely part of an explanation to the declining incidence. The postponement of recurrent strokes on the other hand might well be the result of a more effective secondary treatment with platelet inhibitors and statins as well as a greater awareness of risks associated with atrial fibrillation with a subsequent increased search for atrial fibrillation in ischemic stroke patients and a more widespread use of prophylactic anticoagulant treatment.

Since we know that the population has become older and survival from stroke has improved during the past two decades, the decline in prevalence means that the decline in incidence rate of stroke has been large enough to compensate for the other two factors. Our prevalence proportion is generally lower than in previous studies [25–27]. However, different definitions of stroke prevalence (duration and accumulation period) make direct comparisons impossible. Our prevalence proportion was around 3.5% in men and slightly less than 3% in women, using a definition of prevalence as the proportion alive with a history of any stroke up to 7 years back in time.

As for the decline in absolute number of incident stroke cases, a study from Canada found an increase in hospital admissions between 2003 and 2012 (despite declining rates, due to population growth). While their study was based on hospital admissions only, we take into account fatal strokes as well, occurring outside of hospital. However, since it’s possible that over time, more individuals reach hospitals instead of die outside of hospitals, our decline may hide an increase in hospital admissions even if total number of strokes decrease. But restricting to hospitalized patients only but still resulted in a decline in absolute number of strokes from year 2000 and onwards.

Our calculations are based on Swedish data. Even if the level of the incidence rates are likely to differ between countries we see no reason why the stroke trends observed in Sweden should be very different from those in other comparable countries with similar health care, but we are eager and curious to see our results be repeated in other populations.

### Strengths and limitations

The strength of this study is its size and the non-selected, nationwide information about hospital admissions and deaths from stroke and detailed and precise data about the population at risk [28, 29]. Since health care is publically available and almost free of charge in Sweden it means that selection bias is a non-issue. Even if hospital-based data have the disadvantage that they include only those cases that lead to hospitalization, certain diseases, for example stroke, are well suited to study based on registers of hospital admissions since almost all events result in a hospital admission (or death). Cases of stroke can thus be identified by combining information about hospital admissions and death [30]. Yet, discrepancies from the true number may still occur due to several reasons such as; i) all stroke patients do not seek hospital care, ii) some present with atypical symptoms, iii) the diagnostic criteria that are used at hospitals do not have 100% sensitivity and specificity, iv) on some instances the diagnosis that is made does not meet the criteria, v) coding errors occur in the medical records. Despite this, studies have shown that hospital admissions and causes of death can be combined to cases of stroke with reasonable accuracy [14, 31, 32], and we believe that the limitations of some misclassification of strokes would not affect the trends found in this study. We defined recurrent stroke as a stroke occurring at least 28 days from the previous stroke. This means that we may miss out early recurrent strokes occurring within the 28 days of a previous stroke. However, we see no reason why the trend of such strokes would be any different from the incidence of first, all or 28 days recurrent, even if the rate of recurrent stroke may be somewhat underestimated in our study in comparison from studies that do not use the 28 days definition.

## Conclusions

The decline in recurrence of stroke has been as strong as the decline in the incidence of first stroke, indicating that both primary and secondary prevention have been successful for reducing stroke incidence and burden of stroke on society. Our result also disproves that recurrence of stroke would have increased when survival from first stroke has increased. Despite an ageing population, the prevalence proportion of stroke has declined since year 2000 because of declining incidence rates of stroke large enough to compensate for improved survival and an older population. Based on the development the past 20 years in Sweden we see no indications of an increased burden of stroke in the near future. However, the lack of improvements in ages above 90 years call for some concern when this age group grows larger.

## Additional files

Additional file 1: **Figure S1a.** Incidence rate of first and all strokes (values on left Y-axis) and incidence rate of second and all recurrent strokes (values on right Y-axis). Values presented over the period 1994 to 2014 for men and women aged 60–64. **Figure S1b.** Incidence rate of first and all strokes (values on left Y-axis) and incidence rate of second and all recurrent strokes (values on right Y-axis). Values presented over the period 1994 to 2014 for men and women aged 65–69. **Figure S1c.** Incidence rate of first and all strokes (values on left Y-axis) and incidence rate of second and all recurrent strokes (values on right Y-axis). Values presented over the period 1994 to 2014 for men and women aged 70–74. **Figure S1d.** Incidence rate of first and all strokes (values on left Y-axis) and incidence rate of second and all recurrent strokes (values on right Y-axis). Values presented over the period 1994 to 2014 for men and women aged 75–79. **Figure S1e.** Incidence rate of first and all strokes (values on left Y-axis) and incidence rate of second and all recurrent strokes (values on right Y-axis). Values presented over the period 1994 to 2014 for men and women aged 80–84. Figure 1f**.** Incidence rate of first and all strokes (values on left Y-axis) and incidence rate of second and all recurrent strokes (values on right Y-axis). Values presented over the period 1994 to 2014 for men and women aged 85–89. Figure 1g**.** Incidence rate of first and all strokes (values on left Y-axis) and incidence rate of second and all recurrent strokes (values on right Y-axis). Values presented over the period 1994 to 2014 for men and women aged 90–94. Figure 1h**.** Incidence rate of first and all strokes (values on left Y-axis) and incidence rate of second and all recurrent strokes (values on right Y-axis). Values presented over the period 1994 to 2014 for men and women aged 95–104. (ZIP 2280 kb)

Additional file 2: **Figure S2.** Absolute number of incident stroke events. (PNG 7 kb)

Additional file 3: **Figure S3a.** Incidence rate of first and all strokes (values on left Y-axis) and incidence rate of second and all recurrent strokes (values on right Y-axis), Ischemic stroke. Values presented over the period 1994 to 2014 for men and women aged 60–104 (age adjusted). **Figure S3b.** Incidence rate of first and all strokes (values on left Y-axis) and incidence rate of second and all recurrent strokes (values on right Y-axis), Haemorrhagic stroke. Values presented over the period 1994 to 2014 for men and women aged 60–104 (age adjusted). (ZIP 285 kb)

## Abbreviations

CDR: Cause of Death Register
ICD: The International Statistical Classification of Diseases and Related Health Problems
NPR: National Patient Register
