# Supplementary figures and images for: Temporal trends in incidence, recurrence and prevalence of stroke in an era of ageing populations, a longitudinal study of the total Swedish population
Source: BMC Geriatr. 2019 Feb 4;19:31. doi: 10.1186/s12877-019-1050-1 (PMC6360781; doi:10.1186/s12877-019-1050-1)

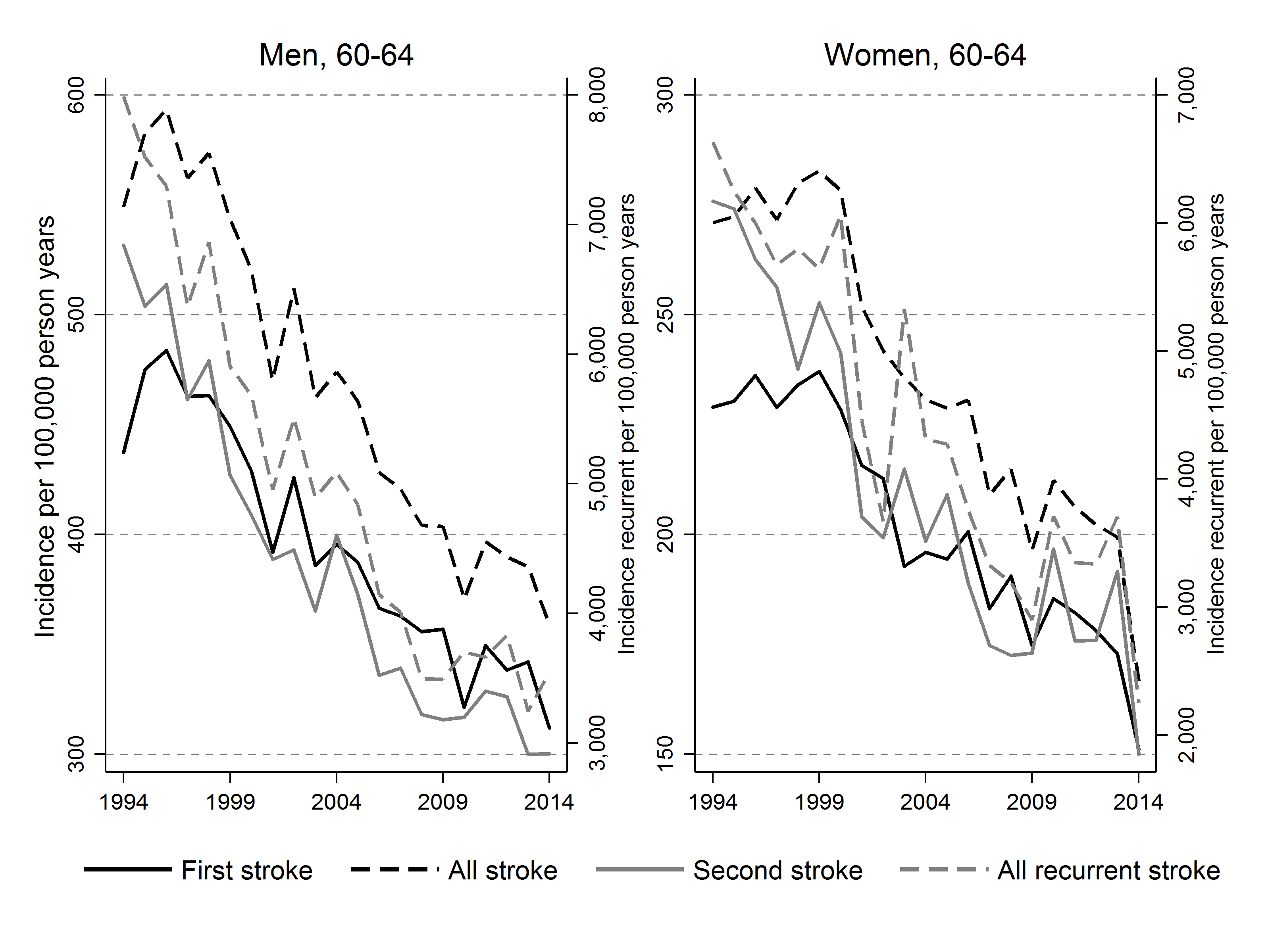

Supplement: Supplementary file 1 — Figure S1a. Incidence rate of first and all strokes (values on left Y-axis) and incidence rate of second and all recurrent strokes (values on right Y-axis). Values presented over the period 1994 to 2014 for men and women aged 60–64. Figure S1b. Incidence rate of first and all strokes (values on left Y-axis) and incidence rate of second and all recurrent strokes (values on right Y-axis). Values presented over the period 1994 to 2014 for men and women aged 65–69. Figure S1c. Incidence rate of first and all strokes (values on left Y-axis) and incidence rate of second and all recurrent strokes (values on right Y-axis). Values presented over the period 1994 to 2014 for men and women aged 70–74. Figure S1d. Incidence rate of first and all strokes (values on left Y-axis) and incidence rate of second and all recurrent strokes (values on right Y-axis). Values presented over the period 1994 to 2014 for men and women aged 75–79. Figure S1e. Incidence rate of first and all strokes (values on left Y-axis) and incidence rate of second and all recurrent strokes (values on right Y-axis). Values presented over the period 1994 to 2014 for men and women aged 80–84. Figure 1f. Incidence rate of first and all strokes (values on left Y-axis) and incidence rate of second and all recurrent strokes (values on right Y-axis). Values presented over the period 1994 to 2014 for men and women aged 85–89. Figure 1g. Incidence rate of first and all strokes (values on left Y-axis) and incidence rate of second and all recurrent strokes (values on right Y-axis). Values presented over the period 1994 to 2014 for men and women aged 90–94. Figure 1h. Incidence rate of first and all strokes (values on left Y-axis) and incidence rate of second and all recurrent strokes (values on right Y-axis). Values presented over the period 1994 to 2014 for men and women aged 95–104. (ZIP 2280 kb) [file 12877_2019_1050_MOESM1_ESM.zip › Additional file 1aR2.png]

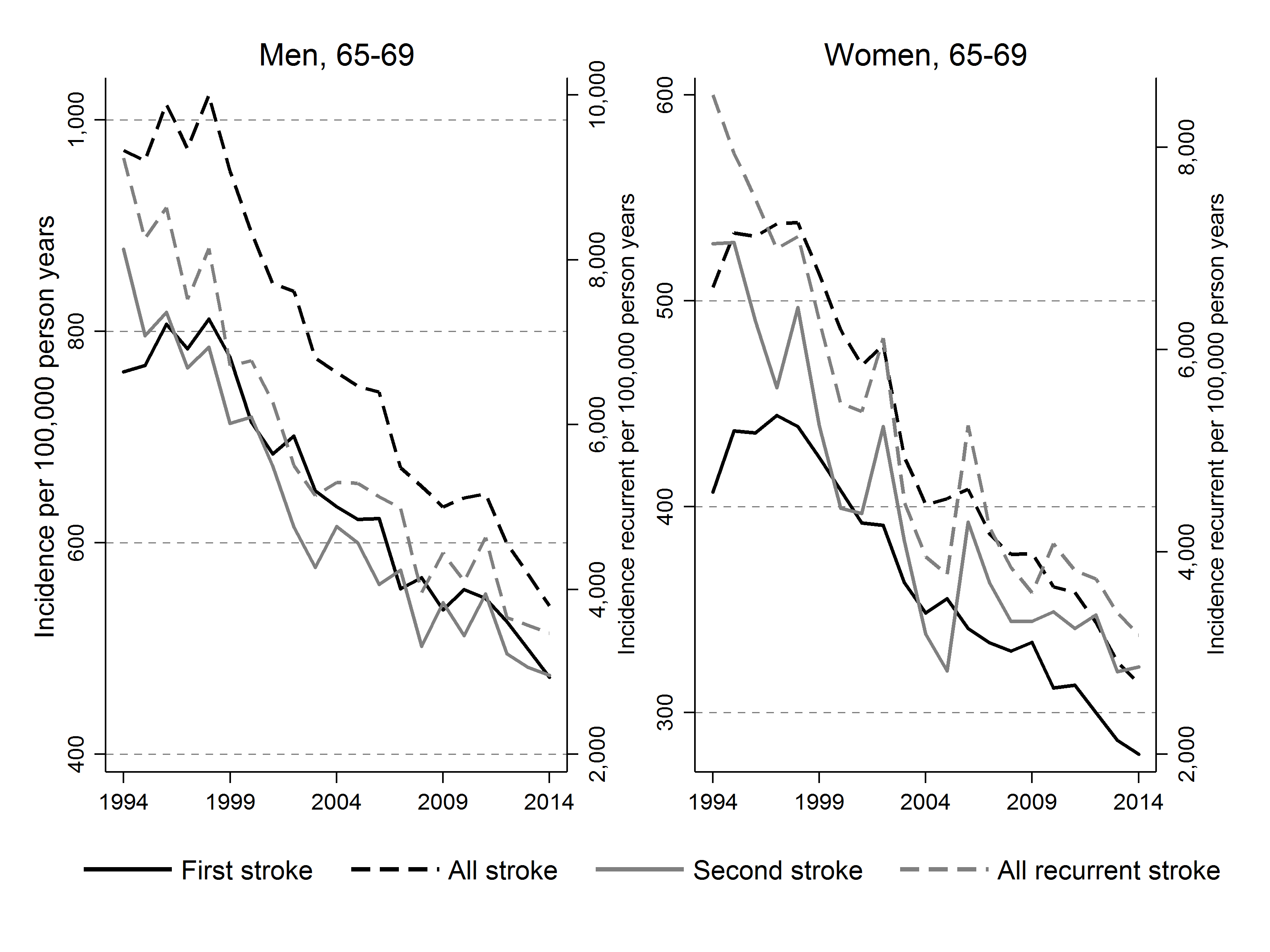

Supplement: Supplementary file 1 — Figure S1a. Incidence rate of first and all strokes (values on left Y-axis) and incidence rate of second and all recurrent strokes (values on right Y-axis). Values presented over the period 1994 to 2014 for men and women aged 60–64. Figure S1b. Incidence rate of first and all strokes (values on left Y-axis) and incidence rate of second and all recurrent strokes (values on right Y-axis). Values presented over the period 1994 to 2014 for men and women aged 65–69. Figure S1c. Incidence rate of first and all strokes (values on left Y-axis) and incidence rate of second and all recurrent strokes (values on right Y-axis). Values presented over the period 1994 to 2014 for men and women aged 70–74. Figure S1d. Incidence rate of first and all strokes (values on left Y-axis) and incidence rate of second and all recurrent strokes (values on right Y-axis). Values presented over the period 1994 to 2014 for men and women aged 75–79. Figure S1e. Incidence rate of first and all strokes (values on left Y-axis) and incidence rate of second and all recurrent strokes (values on right Y-axis). Values presented over the period 1994 to 2014 for men and women aged 80–84. Figure 1f. Incidence rate of first and all strokes (values on left Y-axis) and incidence rate of second and all recurrent strokes (values on right Y-axis). Values presented over the period 1994 to 2014 for men and women aged 85–89. Figure 1g. Incidence rate of first and all strokes (values on left Y-axis) and incidence rate of second and all recurrent strokes (values on right Y-axis). Values presented over the period 1994 to 2014 for men and women aged 90–94. Figure 1h. Incidence rate of first and all strokes (values on left Y-axis) and incidence rate of second and all recurrent strokes (values on right Y-axis). Values presented over the period 1994 to 2014 for men and women aged 95–104. (ZIP 2280 kb) [file 12877_2019_1050_MOESM1_ESM.zip › Additional file 1bR2.png]

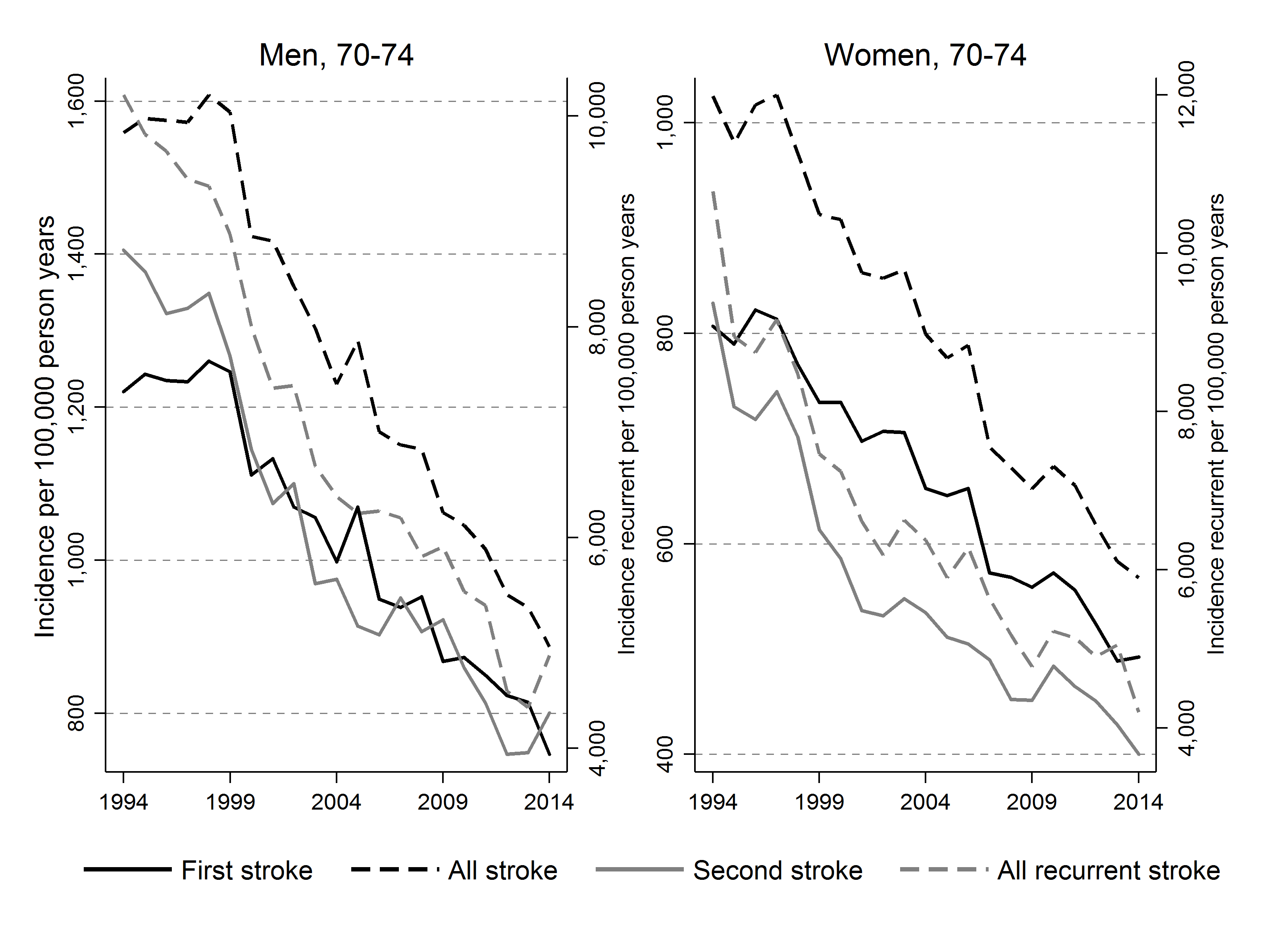

Supplement: Supplementary file 1 — Figure S1a. Incidence rate of first and all strokes (values on left Y-axis) and incidence rate of second and all recurrent strokes (values on right Y-axis). Values presented over the period 1994 to 2014 for men and women aged 60–64. Figure S1b. Incidence rate of first and all strokes (values on left Y-axis) and incidence rate of second and all recurrent strokes (values on right Y-axis). Values presented over the period 1994 to 2014 for men and women aged 65–69. Figure S1c. Incidence rate of first and all strokes (values on left Y-axis) and incidence rate of second and all recurrent strokes (values on right Y-axis). Values presented over the period 1994 to 2014 for men and women aged 70–74. Figure S1d. Incidence rate of first and all strokes (values on left Y-axis) and incidence rate of second and all recurrent strokes (values on right Y-axis). Values presented over the period 1994 to 2014 for men and women aged 75–79. Figure S1e. Incidence rate of first and all strokes (values on left Y-axis) and incidence rate of second and all recurrent strokes (values on right Y-axis). Values presented over the period 1994 to 2014 for men and women aged 80–84. Figure 1f. Incidence rate of first and all strokes (values on left Y-axis) and incidence rate of second and all recurrent strokes (values on right Y-axis). Values presented over the period 1994 to 2014 for men and women aged 85–89. Figure 1g. Incidence rate of first and all strokes (values on left Y-axis) and incidence rate of second and all recurrent strokes (values on right Y-axis). Values presented over the period 1994 to 2014 for men and women aged 90–94. Figure 1h. Incidence rate of first and all strokes (values on left Y-axis) and incidence rate of second and all recurrent strokes (values on right Y-axis). Values presented over the period 1994 to 2014 for men and women aged 95–104. (ZIP 2280 kb) [file 12877_2019_1050_MOESM1_ESM.zip › Additional file 1cR2.png]

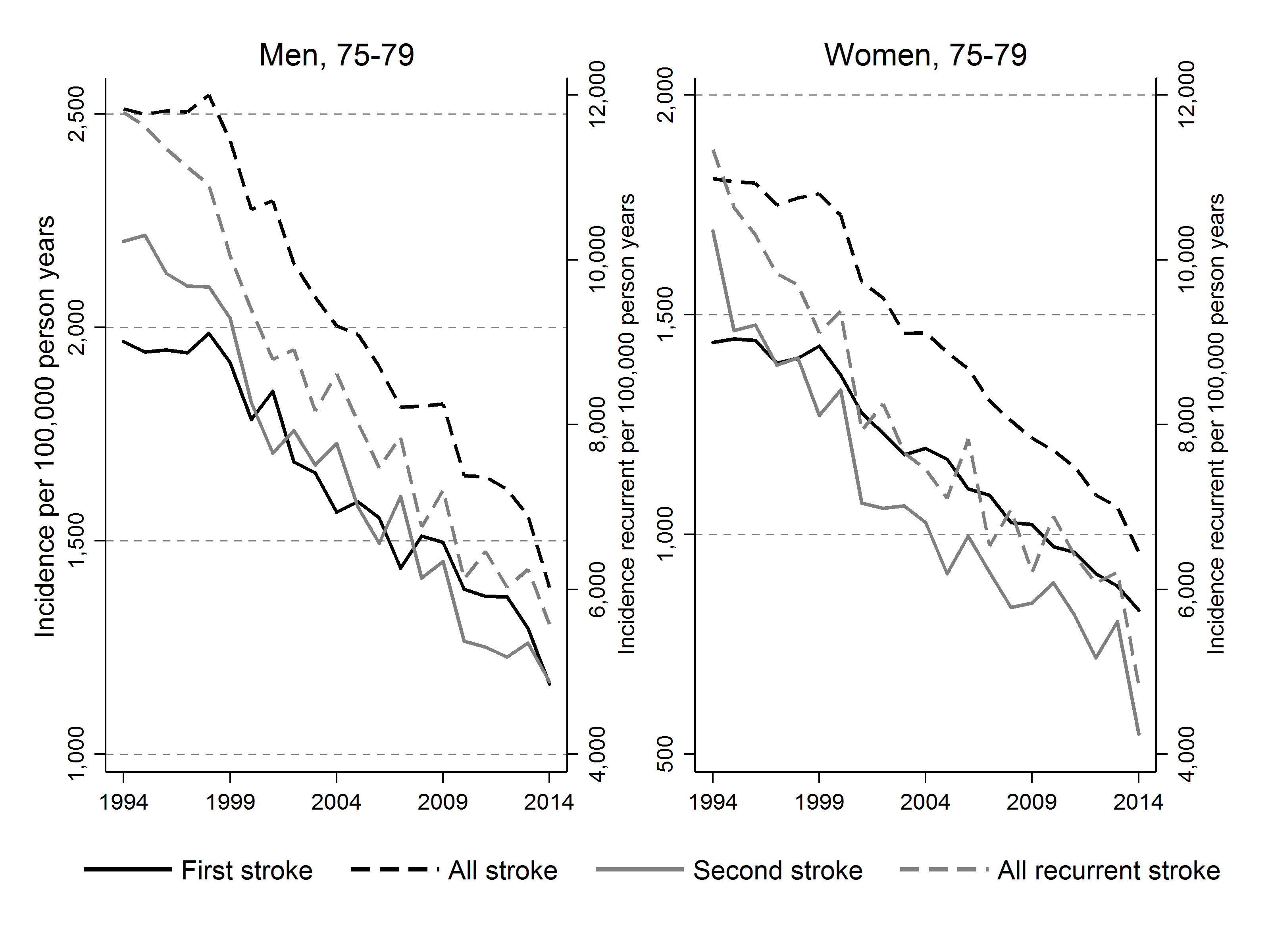

Supplement: Supplementary file 1 — Figure S1a. Incidence rate of first and all strokes (values on left Y-axis) and incidence rate of second and all recurrent strokes (values on right Y-axis). Values presented over the period 1994 to 2014 for men and women aged 60–64. Figure S1b. Incidence rate of first and all strokes (values on left Y-axis) and incidence rate of second and all recurrent strokes (values on right Y-axis). Values presented over the period 1994 to 2014 for men and women aged 65–69. Figure S1c. Incidence rate of first and all strokes (values on left Y-axis) and incidence rate of second and all recurrent strokes (values on right Y-axis). Values presented over the period 1994 to 2014 for men and women aged 70–74. Figure S1d. Incidence rate of first and all strokes (values on left Y-axis) and incidence rate of second and all recurrent strokes (values on right Y-axis). Values presented over the period 1994 to 2014 for men and women aged 75–79. Figure S1e. Incidence rate of first and all strokes (values on left Y-axis) and incidence rate of second and all recurrent strokes (values on right Y-axis). Values presented over the period 1994 to 2014 for men and women aged 80–84. Figure 1f. Incidence rate of first and all strokes (values on left Y-axis) and incidence rate of second and all recurrent strokes (values on right Y-axis). Values presented over the period 1994 to 2014 for men and women aged 85–89. Figure 1g. Incidence rate of first and all strokes (values on left Y-axis) and incidence rate of second and all recurrent strokes (values on right Y-axis). Values presented over the period 1994 to 2014 for men and women aged 90–94. Figure 1h. Incidence rate of first and all strokes (values on left Y-axis) and incidence rate of second and all recurrent strokes (values on right Y-axis). Values presented over the period 1994 to 2014 for men and women aged 95–104. (ZIP 2280 kb) [file 12877_2019_1050_MOESM1_ESM.zip › Additional file 1dR2.png]

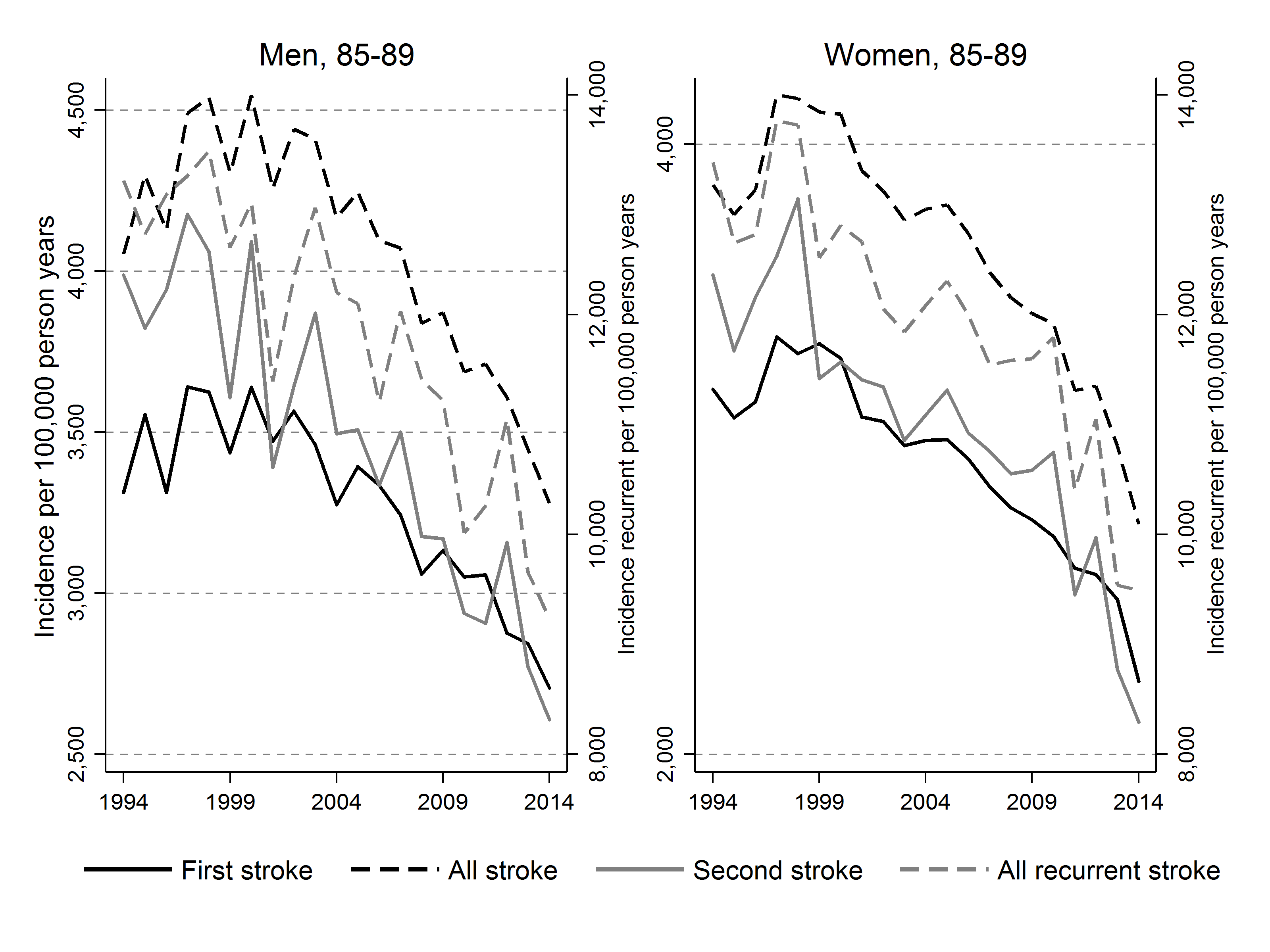

Supplement: Supplementary file 1 — Figure S1a. Incidence rate of first and all strokes (values on left Y-axis) and incidence rate of second and all recurrent strokes (values on right Y-axis). Values presented over the period 1994 to 2014 for men and women aged 60–64. Figure S1b. Incidence rate of first and all strokes (values on left Y-axis) and incidence rate of second and all recurrent strokes (values on right Y-axis). Values presented over the period 1994 to 2014 for men and women aged 65–69. Figure S1c. Incidence rate of first and all strokes (values on left Y-axis) and incidence rate of second and all recurrent strokes (values on right Y-axis). Values presented over the period 1994 to 2014 for men and women aged 70–74. Figure S1d. Incidence rate of first and all strokes (values on left Y-axis) and incidence rate of second and all recurrent strokes (values on right Y-axis). Values presented over the period 1994 to 2014 for men and women aged 75–79. Figure S1e. Incidence rate of first and all strokes (values on left Y-axis) and incidence rate of second and all recurrent strokes (values on right Y-axis). Values presented over the period 1994 to 2014 for men and women aged 80–84. Figure 1f. Incidence rate of first and all strokes (values on left Y-axis) and incidence rate of second and all recurrent strokes (values on right Y-axis). Values presented over the period 1994 to 2014 for men and women aged 85–89. Figure 1g. Incidence rate of first and all strokes (values on left Y-axis) and incidence rate of second and all recurrent strokes (values on right Y-axis). Values presented over the period 1994 to 2014 for men and women aged 90–94. Figure 1h. Incidence rate of first and all strokes (values on left Y-axis) and incidence rate of second and all recurrent strokes (values on right Y-axis). Values presented over the period 1994 to 2014 for men and women aged 95–104. (ZIP 2280 kb) [file 12877_2019_1050_MOESM1_ESM.zip › Additional file 1fR2.png]

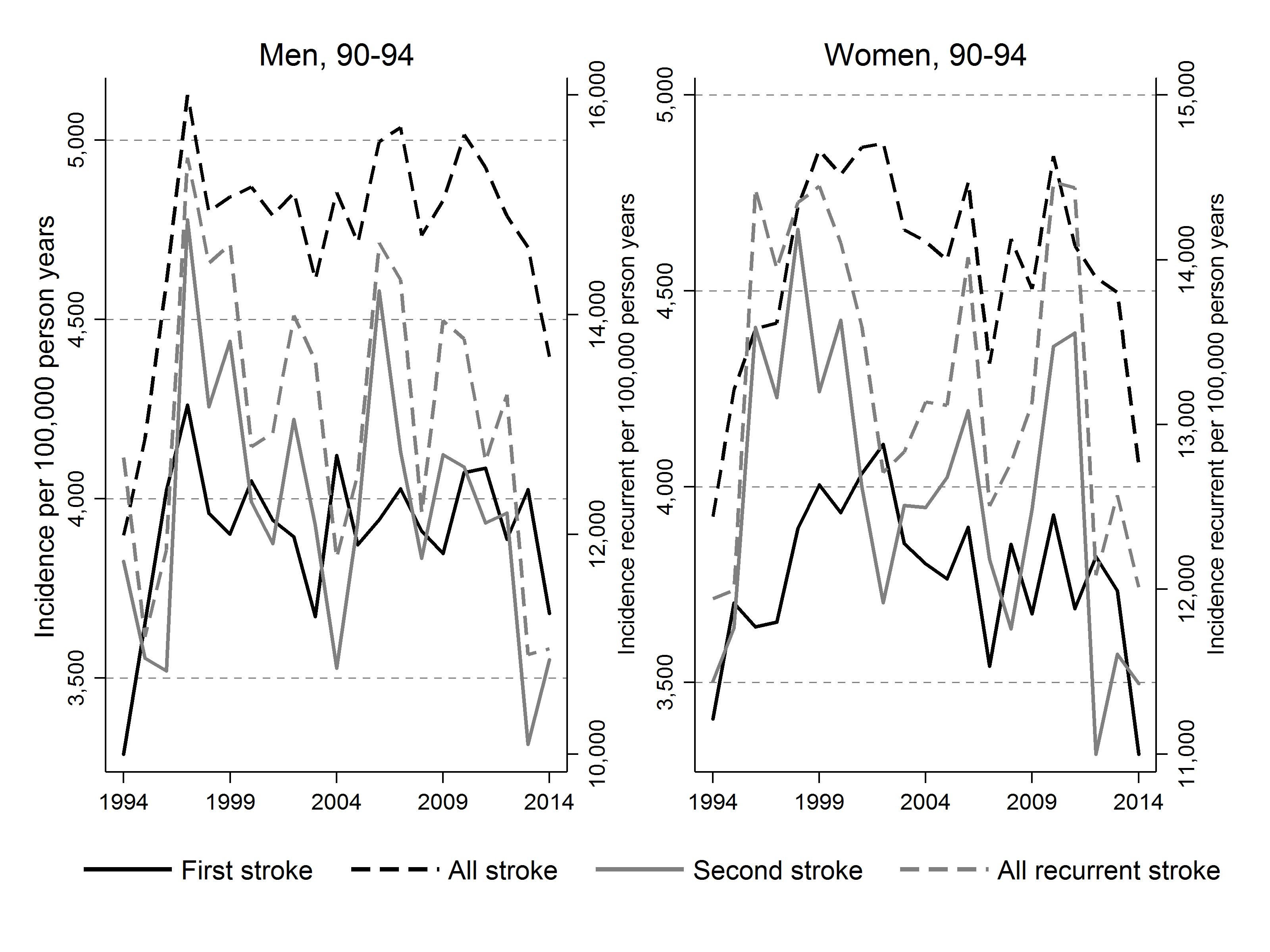

Supplement: Supplementary file 1 — Figure S1a. Incidence rate of first and all strokes (values on left Y-axis) and incidence rate of second and all recurrent strokes (values on right Y-axis). Values presented over the period 1994 to 2014 for men and women aged 60–64. Figure S1b. Incidence rate of first and all strokes (values on left Y-axis) and incidence rate of second and all recurrent strokes (values on right Y-axis). Values presented over the period 1994 to 2014 for men and women aged 65–69. Figure S1c. Incidence rate of first and all strokes (values on left Y-axis) and incidence rate of second and all recurrent strokes (values on right Y-axis). Values presented over the period 1994 to 2014 for men and women aged 70–74. Figure S1d. Incidence rate of first and all strokes (values on left Y-axis) and incidence rate of second and all recurrent strokes (values on right Y-axis). Values presented over the period 1994 to 2014 for men and women aged 75–79. Figure S1e. Incidence rate of first and all strokes (values on left Y-axis) and incidence rate of second and all recurrent strokes (values on right Y-axis). Values presented over the period 1994 to 2014 for men and women aged 80–84. Figure 1f. Incidence rate of first and all strokes (values on left Y-axis) and incidence rate of second and all recurrent strokes (values on right Y-axis). Values presented over the period 1994 to 2014 for men and women aged 85–89. Figure 1g. Incidence rate of first and all strokes (values on left Y-axis) and incidence rate of second and all recurrent strokes (values on right Y-axis). Values presented over the period 1994 to 2014 for men and women aged 90–94. Figure 1h. Incidence rate of first and all strokes (values on left Y-axis) and incidence rate of second and all recurrent strokes (values on right Y-axis). Values presented over the period 1994 to 2014 for men and women aged 95–104. (ZIP 2280 kb) [file 12877_2019_1050_MOESM1_ESM.zip › Additional file 1gR2.png]

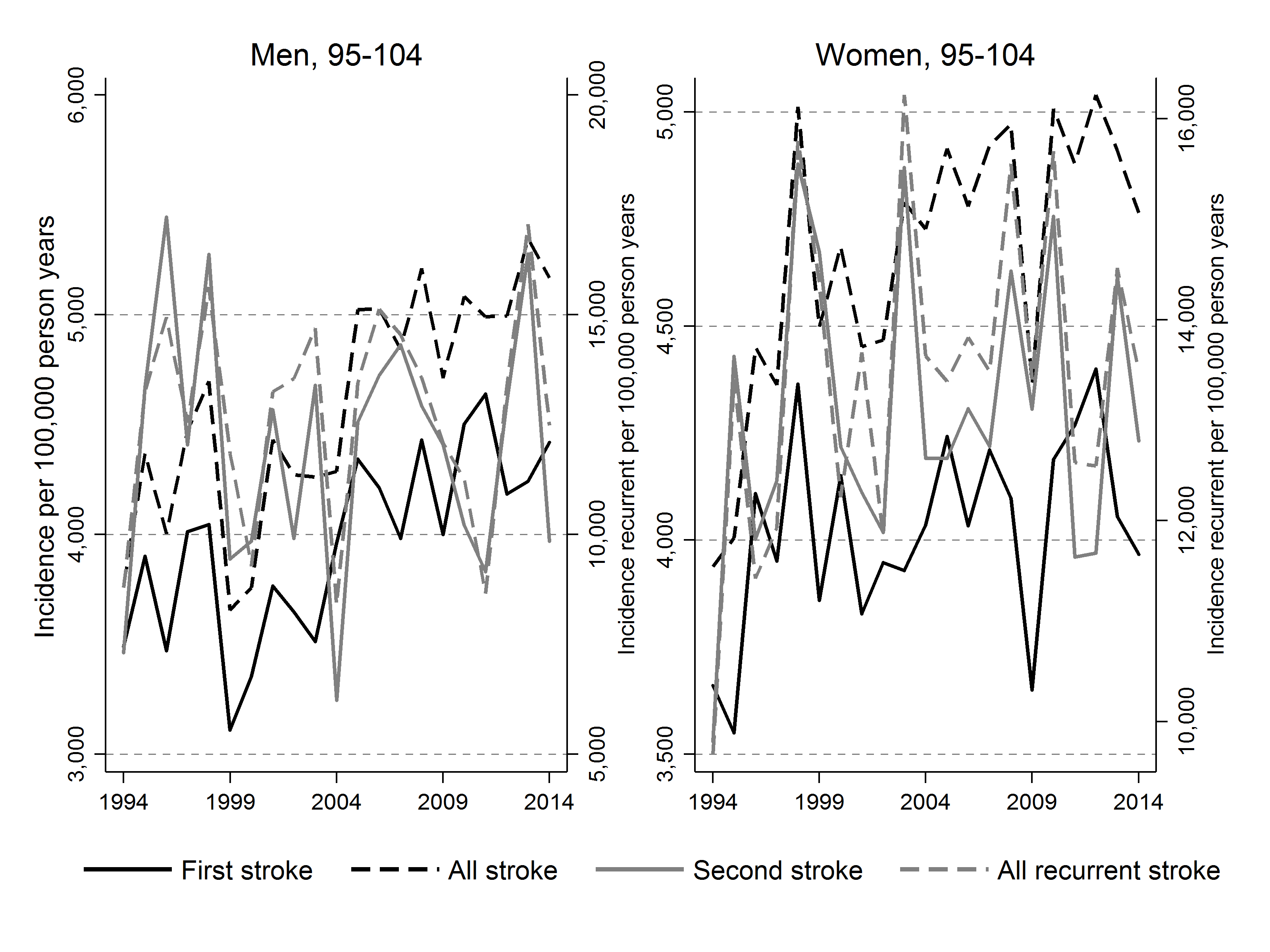

Supplement: Supplementary file 1 — Figure S1a. Incidence rate of first and all strokes (values on left Y-axis) and incidence rate of second and all recurrent strokes (values on right Y-axis). Values presented over the period 1994 to 2014 for men and women aged 60–64. Figure S1b. Incidence rate of first and all strokes (values on left Y-axis) and incidence rate of second and all recurrent strokes (values on right Y-axis). Values presented over the period 1994 to 2014 for men and women aged 65–69. Figure S1c. Incidence rate of first and all strokes (values on left Y-axis) and incidence rate of second and all recurrent strokes (values on right Y-axis). Values presented over the period 1994 to 2014 for men and women aged 70–74. Figure S1d. Incidence rate of first and all strokes (values on left Y-axis) and incidence rate of second and all recurrent strokes (values on right Y-axis). Values presented over the period 1994 to 2014 for men and women aged 75–79. Figure S1e. Incidence rate of first and all strokes (values on left Y-axis) and incidence rate of second and all recurrent strokes (values on right Y-axis). Values presented over the period 1994 to 2014 for men and women aged 80–84. Figure 1f. Incidence rate of first and all strokes (values on left Y-axis) and incidence rate of second and all recurrent strokes (values on right Y-axis). Values presented over the period 1994 to 2014 for men and women aged 85–89. Figure 1g. Incidence rate of first and all strokes (values on left Y-axis) and incidence rate of second and all recurrent strokes (values on right Y-axis). Values presented over the period 1994 to 2014 for men and women aged 90–94. Figure 1h. Incidence rate of first and all strokes (values on left Y-axis) and incidence rate of second and all recurrent strokes (values on right Y-axis). Values presented over the period 1994 to 2014 for men and women aged 95–104. (ZIP 2280 kb) [file 12877_2019_1050_MOESM1_ESM.zip › Additional file 1hR2.png]

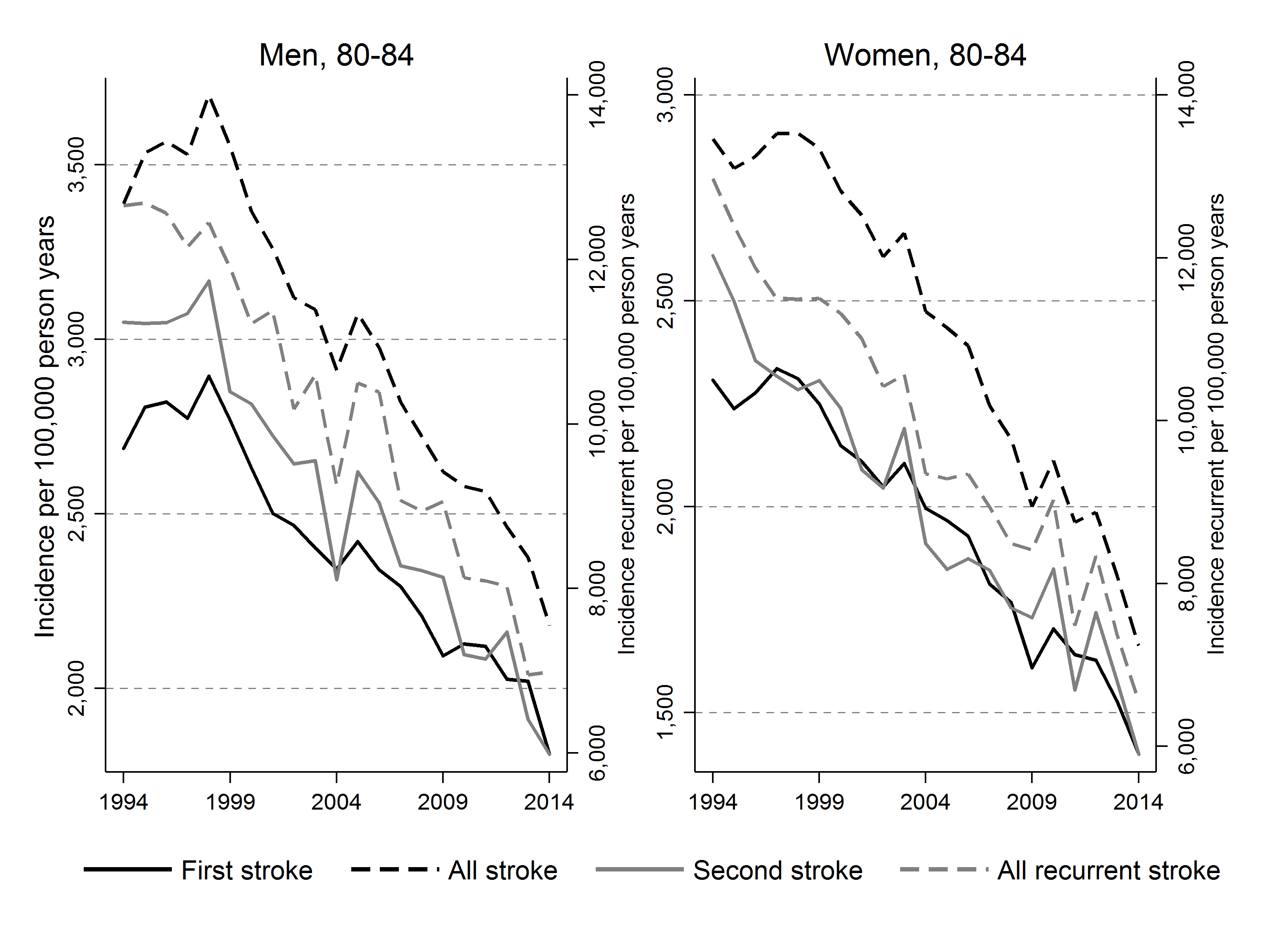

Supplement: Supplementary file 1 — Figure S1a. Incidence rate of first and all strokes (values on left Y-axis) and incidence rate of second and all recurrent strokes (values on right Y-axis). Values presented over the period 1994 to 2014 for men and women aged 60–64. Figure S1b. Incidence rate of first and all strokes (values on left Y-axis) and incidence rate of second and all recurrent strokes (values on right Y-axis). Values presented over the period 1994 to 2014 for men and women aged 65–69. Figure S1c. Incidence rate of first and all strokes (values on left Y-axis) and incidence rate of second and all recurrent strokes (values on right Y-axis). Values presented over the period 1994 to 2014 for men and women aged 70–74. Figure S1d. Incidence rate of first and all strokes (values on left Y-axis) and incidence rate of second and all recurrent strokes (values on right Y-axis). Values presented over the period 1994 to 2014 for men and women aged 75–79. Figure S1e. Incidence rate of first and all strokes (values on left Y-axis) and incidence rate of second and all recurrent strokes (values on right Y-axis). Values presented over the period 1994 to 2014 for men and women aged 80–84. Figure 1f. Incidence rate of first and all strokes (values on left Y-axis) and incidence rate of second and all recurrent strokes (values on right Y-axis). Values presented over the period 1994 to 2014 for men and women aged 85–89. Figure 1g. Incidence rate of first and all strokes (values on left Y-axis) and incidence rate of second and all recurrent strokes (values on right Y-axis). Values presented over the period 1994 to 2014 for men and women aged 90–94. Figure 1h. Incidence rate of first and all strokes (values on left Y-axis) and incidence rate of second and all recurrent strokes (values on right Y-axis). Values presented over the period 1994 to 2014 for men and women aged 95–104. (ZIP 2280 kb) [file 12877_2019_1050_MOESM1_ESM.zip › Additional file1eR2.png]

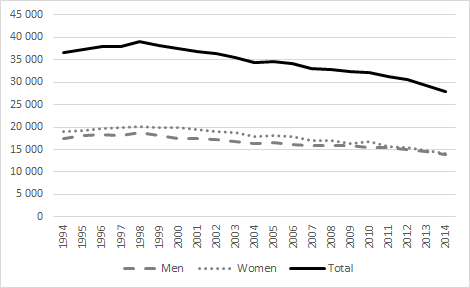

Supplement: Supplementary file 2 — Figure S2. Absolute number of incident stroke events. (PNG 7 kb) [file 12877_2019_1050_MOESM2_ESM.png]

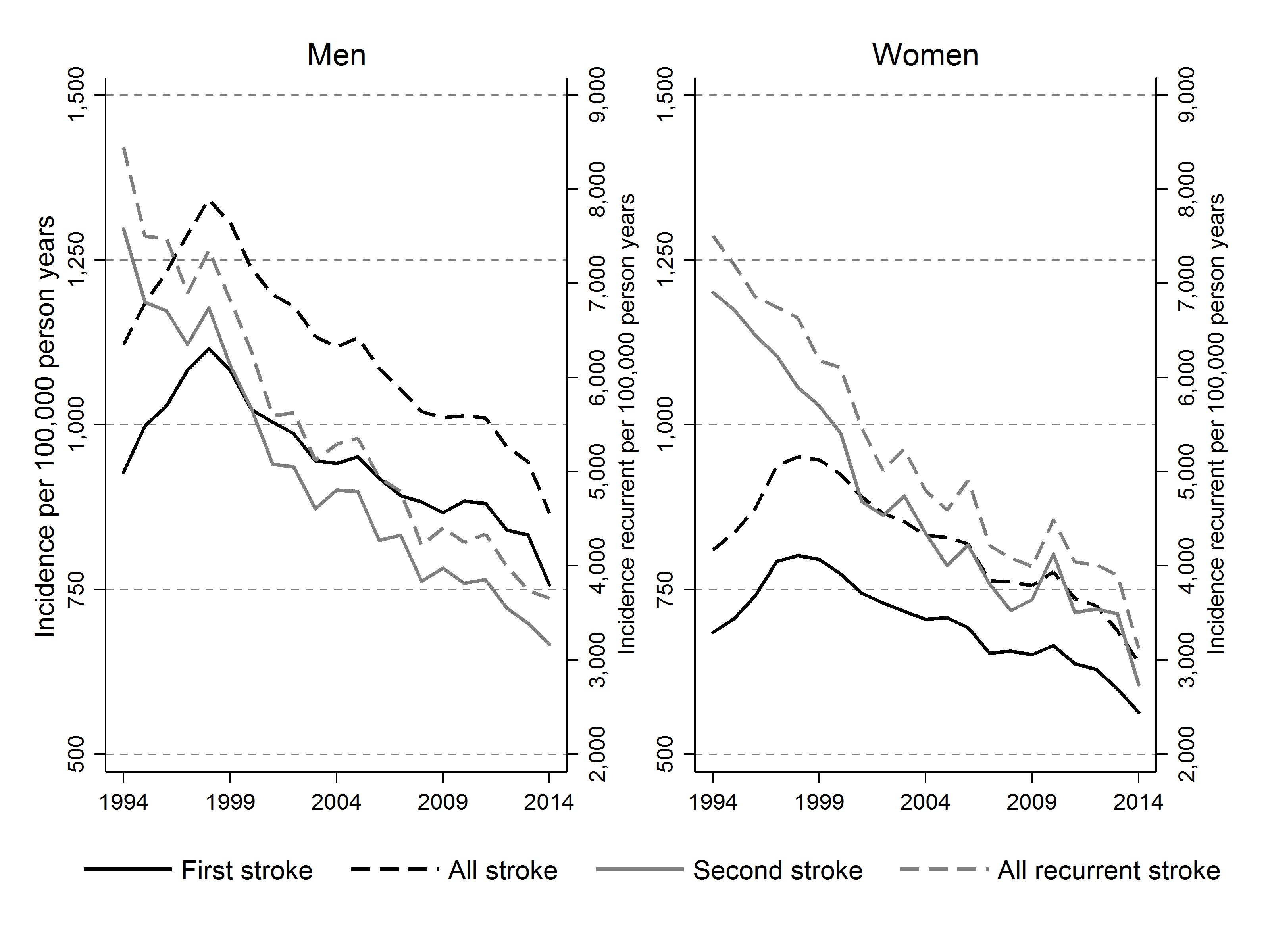

Supplement: Supplementary file 3 — Figure S3a. Incidence rate of first and all strokes (values on left Y-axis) and incidence rate of second and all recurrent strokes (values on right Y-axis), Ischemic stroke. Values presented over the period 1994 to 2014 for men and women aged 60–104 (age adjusted). Figure S3b. Incidence rate of first and all strokes (values on left Y-axis) and incidence rate of second and all recurrent strokes (values on right Y-axis), Haemorrhagic stroke. Values presented over the period 1994 to 2014 for men and women aged 60–104 (age adjusted). (ZIP 285 kb) [file 12877_2019_1050_MOESM3_ESM.zip › Supplementary figure 3aR2.png]

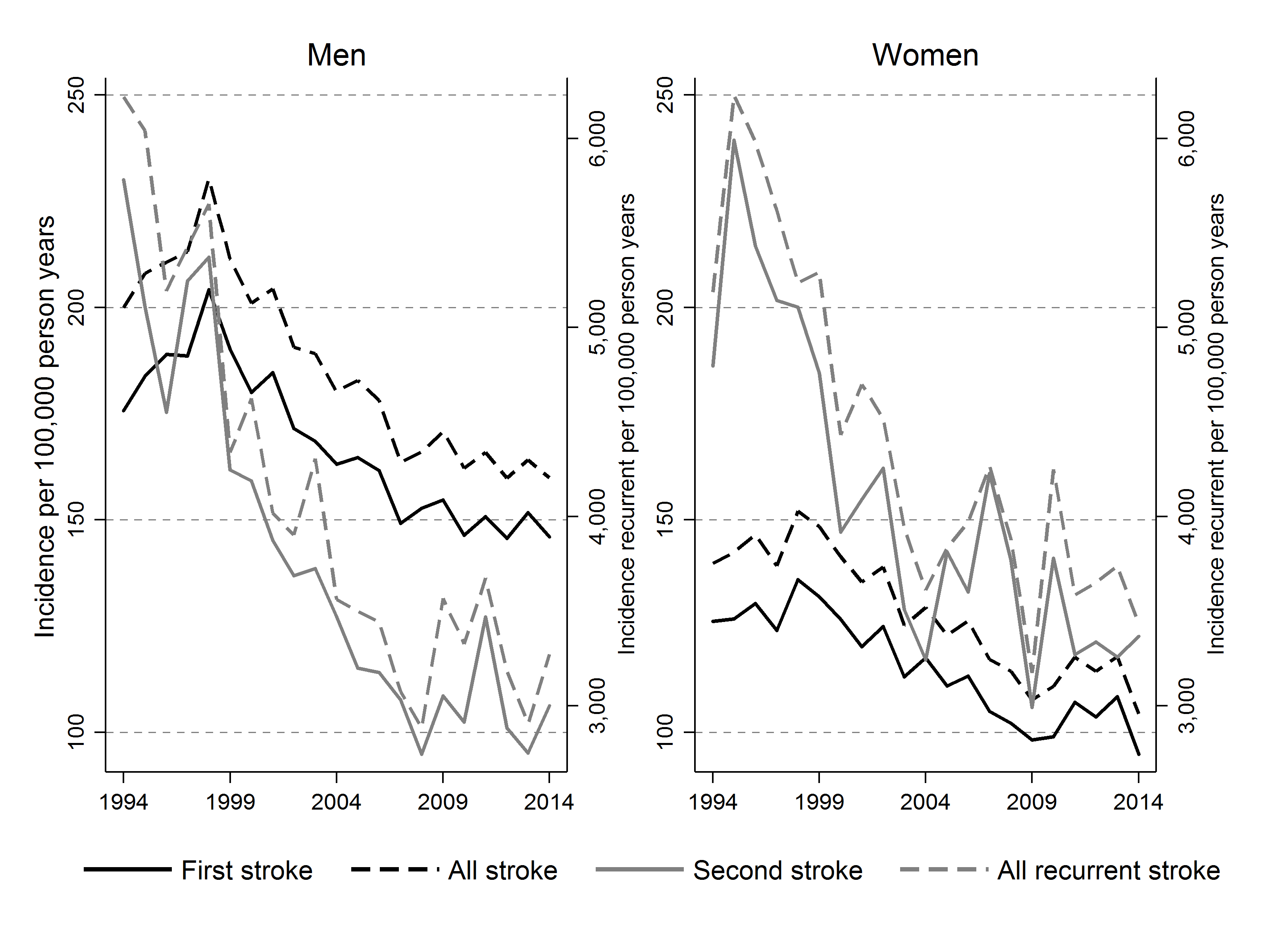

Supplement: Supplementary file 3 — Figure S3a. Incidence rate of first and all strokes (values on left Y-axis) and incidence rate of second and all recurrent strokes (values on right Y-axis), Ischemic stroke. Values presented over the period 1994 to 2014 for men and women aged 60–104 (age adjusted). Figure S3b. Incidence rate of first and all strokes (values on left Y-axis) and incidence rate of second and all recurrent strokes (values on right Y-axis), Haemorrhagic stroke. Values presented over the period 1994 to 2014 for men and women aged 60–104 (age adjusted). (ZIP 285 kb) [file 12877_2019_1050_MOESM3_ESM.zip › Supplementary figure 3b R2.png]
